# Enhancing Skin Delivery and Stability of Vanillic and Ferulic Acids in Aqueous Enzymatically Extracted Glutinous Rice Husk by Nanostructured Lipid Carriers

**DOI:** 10.3390/pharmaceutics15071961

**Published:** 2023-07-16

**Authors:** Sudarat Jiamphun, Wantida Chaiyana

**Affiliations:** 1 Department of Pharmaceutical Science, Faculty of Pharmacy, Chiang Mai University, Chiang Mai 50200, Thailand; jp.plaifah@gmail.com; 2 Research Center of Pharmaceutical Nanotechnology, Faculty of Pharmacy, Chiang Mai University, Chiang Mai 50200, Thailand; 3 Innovation Center for Holistic Health, Nutraceuticals, and Cosmeceuticals, Faculty of Pharmacy, Chiang Mai University, Chiang Mai 50200, Thailand

**Keywords:** *Oryza sativa* var. glutinosa, nanostructure lipid carriers, nano delivery system, vanillic acid, ferulic acid, cosmeceutical, dermal delivery, cellulase, anti-wrinkling

## Abstract

The present study aimed to develop nanostructured lipid carriers (NLCs) and evaluate their effectiveness in enhancing the delivery and stability of vanillic and ferulic acid in the aqueous enzymatic extract of glutinous rice husk using a 0.5% *w*/*w* cellulase solution (CE0.5). NLCs were developed using a high-pressure homogenization technique and characterized for their particle size, polydispersity index, and zeta potential. The entrapment efficiency, physical and chemical stability, release profile, skin permeation, and skin retention of the NLCs loaded with CE0.5 were evaluated. It was observed that NLCs with high entrapment efficiencies efficiently encapsulate and protect both vanillic and ferulic acid, in contrast to a solution. The controlled and sustained release profile of vanillic acid and ferulic acid from NLCs suggests their potential for prolonged and targeted delivery. The findings also demonstrate the superior skin retention capabilities of NLCs without permeation compared to the solution. Notably, NLC2 exhibited the highest delivery into the skin layer, which can be attributed to its smaller particle size (107.3 ± 1.3 nm), enabling enhanced skin penetration. This research highlights the promising application of NLCs in enhancing the delivery and stability of bioactive compounds in cosmetic formulations and related fields.

## 1. Introduction

Rice husk, a ubiquitous byproduct of the rice milling industry, is commonly disposed of through burning, leading to air pollution and posing significant health risks [1]. Rice husk ash, a residual product of the combustion process, consisting of lignocellulosic materials that can be challenging to decompose, is frequently underutilized or directed to landfills, thereby contributing to substantial environmental pollution [2,3]. When rice husk is not disposed of correctly, it can pollute the environment and pose health hazards. Notably, one of the critical issues associated with rice husk is the generation of greenhouse gases. When left to decompose, rice husk releases methane, an impactful greenhouse gas that contributes to global warming [4]. Nonetheless, through appropriate management, rice husk can function as a renewable energy resource. It can be incinerated to produce heat and electricity or transformed into biofuels [5]. Recently, rice husk has gained recognition and prominence in the biofuel industry as a cellulosic feedstock for ethanol biofuel production [6]. Furthermore, rice husk has been explored as a potential ingredient in the cosmetic industry due to its high content of bioactive compounds, such as phenolic acids and flavonoids, which have antioxidant and anti-inflammatory properties [7]. As phenols and flavonoids are indeed naturally occurring compounds present in the original rice husk without requiring any burning or thermal treatment, they can be extracted directly from the raw material. Utilizing rice husk in a way that harnesses these phenols and flavonoids can have the added benefit of reducing air pollution, as it eliminates the need for burning or incineration, which can release harmful pollutants into the atmosphere.

Based on our previous research findings, the aqueous enzymatic extraction of glutinous rice husk using a 0.5% *w*/*w* cellulase aqueous solution (CE0.5) resulted in the identification of vanillic acid (C_8_H_8_O_4_) and ferulic acid (C_10_H_10_O_4_) as prominent constituents. These compounds displayed potent radical scavenging activities and demonstrated an anti-skin wrinkle effect through the inhibition of collagenase (matrix metalloproteinase-1 from *Clostridium histolyticum*: ChC-E.C. 3.4.23.3) and hyaluronidase enzymes (from bovine testicular: E.C.3.2.1.3.5) [8]. As collagenase and hyaluronidase are enzymes that degrade and break down collagen (repeating sequence: (Gly-X-Y)_n_ where X is often Pro, Y is usually 5-hydroxyproline (Hyp), and n may be >300) and hyaluronan (repeating disaccharide units of 2-acetamido-2deoxy-β-d-glucose (GlcNAc) and β-d-glucuronic acid (GlcA) linked 1–4 and 1–3, respectively), inhibition of these enzymes slowed down the degradation of critical structural proteins found abundantly in the skin, resulting in positive effects on the skin, particularly in terms of wrinkling. Collagen, which is the most abundant protein in the human body, is the fundamental structural component of the dermis and plays a crucial role in providing strength, support, and structural integrity to the skin [9]. On the other hand, hyaluronan plays a crucial role in skin hydration by possessing the ability to bind and retain water molecules, promoting moisture retention in the skin tissues, and leading to a reduction in wrinkles and fine lines [10]. These properties make CE0.5 a promising candidate for use in skincare products aimed at slowing down the aging process and preventing skin damage caused by oxidative stress.

In addition to its potential skin benefits, CE0.5 offers the advantage of being an eco-friendly ingredient since it is derived from rice husk, a byproduct of the rice milling industry that would otherwise be discarded. By utilizing this waste material, CE0.5 contributes to sustainable practices and reduces environmental impact. Its use in cosmetics can help reduce waste and promote sustainability in the cosmetic industry. However, more research is needed to develop effective formulations that maximize its benefits. The delivery and stabilization of bioactive compounds, specifically vanillic acid and ferulic acid from CE0.5, play a crucial role in maximizing the efficacy and stability of these compounds in skincare formulations.

Advanced strategies, such as employing nanostructured lipid carriers (NLCs), are actively being explored to improve the skin delivery and stability of bioactive compounds, opening avenues for the development of innovative and effective skincare formulations [11]. NLCs offer a promising approach to overcome the challenges associated with the delivery and stability of bioactive compounds in skincare products [12]. Since NLCs are colloidal lipid-based carriers that consist of a solid lipid matrix and a liquid lipid component, NLCs provided a stable and biocompatible platform for the encapsulation of bioactive compounds [13]. The unique structure of NLCs facilitates the effective integration of both hydrophilic and lipophilic compounds, enhancing their solubility, stability, and permeation into the skin [14]. Despite the initial development of NLCs for lipophilic drug delivery, they have recently gained recognition as suitable carriers for hydrophilic drugs via the incorporation of surfactants or co-surfactants [15]. NLCs have been reported to provide the most effective means of facilitating the transport of hydrophilic drugs across the skin barrier compared to lipid emulsion and solid lipid nanoparticles (SLN), respectively [16]. Moreover, NLCs protect bioactive compounds from degradation, oxidation, and environmental factors, thus extending their shelf life and enhancing their therapeutic potential [17]. The small particle size of NLCs allows for enhanced skin penetration and targeted delivery to specific skin layers, ensuring maximum efficacy. This is particularly beneficial considering that collagenase is naturally found in the dermis, while hyaluronidase is found in both the dermis and epidermis layers. By utilizing NLCs as carriers, the skin delivery and stability of bioactive compounds can be significantly improved, offering a promising approach to overcome the limitations and unlock the full potential of these compounds in skincare formulations [18].

Therefore, the present study aimed to develop NLCs and investigate their effectiveness in improving the delivery and stability of vanillic acid and ferulic acid in CE0.5 for potential application in skincare formulations.

## 2. Materials and Methods

### 2.1. Chemical Materials

Cellulases (1,4-(1,3;1,4)-/3-D-glucan 4-glucanohydrolase, E.C.3.2.1.4, ≥0.3 units/mg solid, optimal pH = 6.5, optimal temperature = 50–55 °C) from *Aspergillus niger*, sodium phosphate (NaH_2_PO_4_), disodium phosphate (Na_2_HPO_4_), and acetic acid were analytical grade, purchased from Sigma-Aldrich (St. Louis, MO, USA). Acetonitrile was HPLC grade, purchased from Sigma-Aldrich (St. Louis, MO, USA). Cetyl alcohol, *Oryza sativa* (rice) bran oil, Polysorbate 80 (Tween^®^ 80, NamSiang Co., Ltd., Bangkok, Thailand), decyl glucoside (Plantacare^®^ 2000, BASF Personal Care and Nutrition GmbH, Monheim, Germany), and sodium cocoyl hydrolyzed pea protein (Coco Pea.Soft^®^, Sinerga S.p.A., Varese, Italy) were cosmetic grade, purchased from NamSiang Co., Ltd. (Bangkok, Thailand).

### 2.2. Plant Materials and Preparation of Glutinous Rice Husk Extract by Aqueous Enzymatic Extraction

Glutinous rice husk from rice milling was obtained from Chiang Mai Phon Suriya Rice Mill, Co., Ltd., Chiang Mai, Thailand. The plant sample was identified and authenticated as *O. sativa* L. var. Niaw San-Pah-Tawng, of which the voucher specimen was kept under number 0023305 at the herbarium, Department of Pharmaceutical Science, Faculty of Pharmacy, Chiang Mai University.

The glutinous rice husk was ground into powder and extracted by aqueous enzymatic extraction using 0.5% *v*/*v* cellulase enzyme aqueous solution adjusted to pH 6.5 at a temperature of 50 °C for 24 h following the method of Jiamphun and Chaiyana (2022) [8]. After removing the glutinous rice husk residues by vacuum filtration through Whatman No. 1 filter paper using a Buchner funnel, the extracting liquid was removed by a freeze-dryer (FreeZone 4.5 model 7750031, Labconco, Kansas, MO, USA). The glutinous rice husk extract (CE0.5), with a yield of 1.9 ± 0.1% *w*/*w* [8], was kept in a well-closed container until the further experiments.

### 2.3. Development of Nanostructured Lipid Carriers (NLCs)

NLCs were developed by using high-pressure homogenization. The pre-emulsion (200 mL) was premixed for 1 min using a high-performance dispersing instrument (Ultra-Turrax T 25 disperser, IKA company, Königswinter, Germany) at 8000 rpm and was then passed through the high-pressure homogenizer (APV-2000, SPX Co., Charlotte, NC, USA) set at 500 Pas for 5 cycles [19,20]. Surfactant type, surfactant quantity, solid lipid amount, and the ratio of solid lipid to liquid lipid were all explored as determinants in the production of NLCs. In the NLC development, cetyl alcohol was employed as a solid lipid and rice bran oil as a liquid lipid, while several surfactants were used, including Tween^®^ 80, Plantacare^®^ 2000, and Coco Pea.Soft^®^.

### 2.4. Characterization of NLCs

NLCs were characterized for particle size, polydispersity index (PDI), and zeta potential using Malvern zetasizer Nano ZS (Malvern Instruments Ltd., Malvern, UK). The NLC samples were diluted with DI water at a 1:100 ratio prior to the characterizations to avoid particle aggregation and reduce the proximity of the particles to each other. Moreover, it allows individual particles to be more uniformly dispersed in the measurement medium. In the present study, only DI water was used as a dilution medium to avoid the potential effects of ions on the zeta potential measurement. The experiments were repeated three times, with the findings presented as mean and standard deviation (S.D.).

### 2.5. Stability Test of NLCs

The stability of NLCs was evaluated under 8 cycles of heating–cooling conditions [20]. In each cycle, the NLCs were held at 45 °C for 24 h and then changed to 4 °C for 24 h. The physical appearance, particle size, PDI, and zeta potential were characterized. The experiments were repeated three times, with the findings presented as mean and S.D.

### 2.6. Development of Glutinous Rice-Husk-Extract-Loaded NLCs

The NLCs with favorable characteristics with small droplet size, narrow PDI, appropriate zeta potential values, and stability following the stability test were chosen for the incorporation of CE0.5. The glutinous rice-husk-extract-loaded NLCs were prepared using a high-pressure homogenizer, following the previously described method. Subsequently, the particle size, polydispersity index (PDI), and zeta potential of the glutinous rice-husk-extract-loaded NLCs were characterized, as described above.

### 2.7. Entrapment Efficiency Determination of Glutinous Rice-Husk-Extract-Loaded NLCs

The entrapment efficiency of glutinous rice-husk-extract-loaded NLCs was determined using the method described by Shah et al. (2016) with some modifications [21]. In brief, the NLCs were subjected to centrifugation set at 10,000 rpm for 1 h using a Thermo Fisher Scientific centrifuge (Sorvall ST16R, Waltham, MA, USA) to separate the unentrapped extract from the NLCs. The supernatant containing the unentrapped extract was collected and analyzed by HPLC. The entrapment efficiency was calculated using the following equation:

Entrapment efficiency (%) = (Wt − Wu)/Wt × 100

where Wt is total extract and Wu is unentrapped extract. The results were expressed as mean ± SD from triplicate measurements.

### 2.8. Stability Test of Glutinous Rice-Husk-Extract-Loaded NLCs

#### 2.8.1. Storage Condition

The glutinous rice-husk-extract-loaded NLCs were subjected to 8 cycles of heating–cooling condition and kept at various temperatures for 90 days. The physical and chemical stability were investigated after the heating–cooling condition as well as storage for 30, 60, and 90 days at 4 °C, 45 °C, and room temperature.

#### 2.8.2. Physical Stability Determination of Glutinous Rice-Husk-Extract-Loaded NLCs

The physical stability in terms of physical appearance, particle size, PDI, and zeta potential of glutinous rice-husk-extract-loaded NLCs was evaluated after various storage conditions as mentioned above. Each investigation, except the physical appearance, was repeated three times, with the findings presented as mean and S.D.

#### 2.8.3. Chemical Stability Determination of Glutinous Rice-Husk-Extract-Loaded NLCs

The chemical stability of glutinous rice-husk-extract-loaded NLCs in terms of ferulic acid and vanillic acid content was investigated following various storage conditions as mentioned above. The quantity of ferulic acid and vanillic acid were investigated by high-performance liquid chromatography (HPLC) following the previous study of Jiamphun and Chaiyana (2022) [8]. Prior to the investigation, the NLC was mixed with ethanol at a ratio of 1:1 and filtered through a 0.45 µm nylon filter membrane. Supelcosil LC-18 column (250 mm 4.6 mm, 5 µm; Supelco Analytical, Bellefonte, PA, USA) at a fixed temperature of 38 °C was used as a stationary phase [8,22], whereas a combination of acetonitrile and 0.1% *v*/*v* acetic acid aqueous solution in a 20:80 ratio was used as a mobile phase. Each sample was eluted through the stationary phase at a flow rate of 1 mL/min and detected using a UV detector set to 280 nm. Each investigation was repeated three times, with the findings presented as mean and S.D.

### 2.9. Release Study of Glutinous Rice-Husk-Extract-Loaded NLCs

The release study of glutinous rice husk extract from NLCs and aqueous solutions was investigated following the method of Chaiyana et al. (2020). In brief, a 2.5 × 2.5 cm dialysis bag (Cellu Sep, nominal molecular weight cut off 6000–8000, Membrane Filtration Products, Seguin, TX, USA) was filled with 1 mL of the tested formulations. Each dialysis bag was then immersed in a phosphate-buffered saline (PBS) solution with a pH of 5.5, which was continually stirred using a multi-position magnetic stirrer (AM4, EOS Scientific Co., Ltd., Bangkok, Thailand) set to 32 °C. The PBS medium was removed at varied intervals of 1, 2, 4, 8, and 24 h to determine the quantity of released ferulic acid and vanillic acid from the glutinous rice-husk-extract-loaded NLCs by using HPLC as described above. Once the PBS medium was withdrawn, it was instantly replaced with an equal amount of new PBS medium. Each investigation was repeated three times, with the findings presented as mean and S.D.

### 2.10. Skin Permeation and Skin Retention of Glutinous Rice-Husk-Extract-Loaded NLCs

#### 2.10.1. Skin Preparation

The premature piglets, which were born stillborn and had naturally passed away, were acquired from a local farm in Chiang Mai, Thailand, shortly after their demise. The ethical issues associated with the use of animal skin in preliminary permeation screenings can be circumvented if the skin is obtained from animals that have died naturally [23]. The skin was removed from the stillborn piglet’s flank area and washed with PBS pH 5.5. The subcutaneous fat was trimmed off and stored at −20 °C until further used.

#### 2.10.2. Skin Permeation Determination

The skin permeation of glutinous rice husk extract from NLCs and aqueous solutions was examined using Franz diffusion cells as described by Chaiyana et al. (2013) [24]. The stillborn piglet’s flank skin was used as a skin membrane. Each sample with an exact amount of 500 µL was deposited in the donor compartment. The blank NLC formulations without CE0.5 were served as a control. PBS pH 7.4 maintained at 37 °C and stirred consistently using a magnetic stirrer set at 100 rpm was used as a receptor medium, which was withdrawn at various time periods, including 1, 2, 4, 8, and 24 h. The quantity of permeated ferulic acid and vanillic acid from the glutinous rice-husk-extract-loaded NLCs through the skin was determined by HPLC as described above. Once the PBS medium was withdrawn, it was instantly replaced with an equal amount of new PBS medium. Each investigation was repeated three times, with the findings presented as mean and S.D.

#### 2.10.3. Skin Retention Determination

The skin from a previous skin permeation study was collected to evaluate the quantity of ferulic acid and vanillic acid from glutinous rice-husk-extract-loaded NLCs and aqueous solutions that were maintained in the skin layer. Prior to the investigation, the skin underwent a cleansing process using flowing tap water from the faucet. The skin retention was studied using an approach previously described by Chaiyana et al. (2013) [24]. The cleansed skin was homogenized in a combination of ethanol and DI water in a ratio of 1:1 in order to extract ferulic acid and vanillic acid, which were the major bioactive components of the glutinous rice husk extracts. The quantity of both compounds which are preserved in the epidermal layer was determined using the previously established HPLC technique. Each investigation was repeated three times, with the findings presented as a mean and S.D.

### 2.11. Statistical Analysis

The data were presented as a mean and S.D. GraphPad Prism (version 8.0, GraphPad Software) was used to perform paired sample *t*-test and one-way analysis of variance (ANOVA) to determine statistical significance. The level for statistical significance was established at *p* < 0.05.

## 3. Results and Discussion

### 3.1. Nanostructured Lipid Carriers (NLCs)

**Figure 1:**
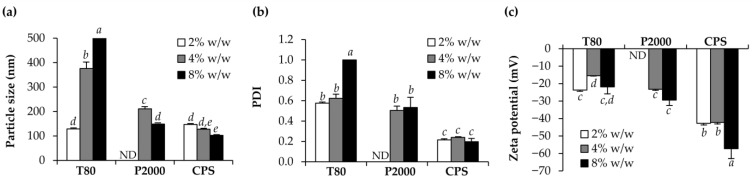
Particle size (**a**), polydispersity index (PDI) (**b**), and zeta potential (**c**) of NLCs developed using different types of surfactants, including Tween^®^ 80 (T80), Plantacare^®^ 2000 (P2000), and Coco Pea.Soft^®^ (CPS), at the various concentration of 2, 4 and 8% *w*/*w*. Different letters, *a*, *b*, *c*, *d* and *e*, denote significant differences in the particle size, PDI, and zeta potential of NLCs analyzed using one-way ANOVA with post hoc Tukey test (*p* < 0.05). ND = not determined.

In the development of NLCs, cetyl alcohol was used as a solid lipid, while Oryza sativa (rice) bran oil was used as a liquid lipid. The effects of different surfactant types and amounts are demonstrated in Figure 1. Polysorbate 80 (Tween^®^ 80) and decyl glucoside (Plantacare^®^ 2000) were the nonionic surfactants used in this investigation. An anionic plant-origin surfactant recommended as a skin conditioner and anti-irritant, sodium cocoyl hydrolyzed pea protein (Coco Pea.Soft^®^), was also employed. It was obviously observed that Plantacare^®^ 2000 required a high concentration of at least 4% *w*/*w* to develop the NLC and no NLC formation when 2% *w*/*w* was applied. The likely explanation for the higher concentration requirement of Plantacare^®^ 2000 in the formulation compared to Tween^®^ 80 may be due to the higher critical micelle concentration (CMC) of Plantacare^®^ 2000 (2.2 × 10^−3^ M [25]) in comparison to Tween^®^ 80 (1.89 × 10^−5^ M [26]). A higher Plantacare^®^ 2000 concentration results in a lower NLC particle size. The results were in line with those of Coco Pea.Soft^®^, in which the higher concentration also led to a lower NLC particle size. As the surfactant reduces the interfacial tension and stabilizes newly generated interfaces during the homogenization process, the creation of smaller NLC particles was generally observed in the system with higher surfactant concentration [27,28,29]. In contrast, a higher Tween^®^ 80 concentration resulted in a larger NLC particle size. The most plausible reason was that Tween^®^ 80 coated on the surface of nanoparticles and therefore increased the NLC particle size when its quantity surpassed a certain level [30]. The results were in line with the previous study of How et al. (2012), who reported a larger NLC particle size when the concentration of Tween^®^ 80 increased from 1 to 4% *w*/*w* [30]. On the other hand, different types of surfactant also affected the PDI of NLC particles. Coco Pea.Soft^®^ yielded the narrowest PDI of around 0.2, which represented a narrow and homogeneous distribution. The higher concentration of surfactant did not affect the PDI, except in the systems of Tween^®^ 80. The explanation was also due to the non-specific adsorption of Tween^®^ 80 on the NLC surfaces, which resulted in an increased PDI [30]. Apart from the narrowest PDI, Coco Pea.Soft^®^ also yielded the NLCs with the most significantly pronounced minus zeta potential values (*p* < 0.05). As a result, Coco Pea.Soft^®^ tended to produce stable NLCs since the zeta potential evaluations provide predictions on the storage stability of submicron colloidal dispersion [31].

**Figure 2:**
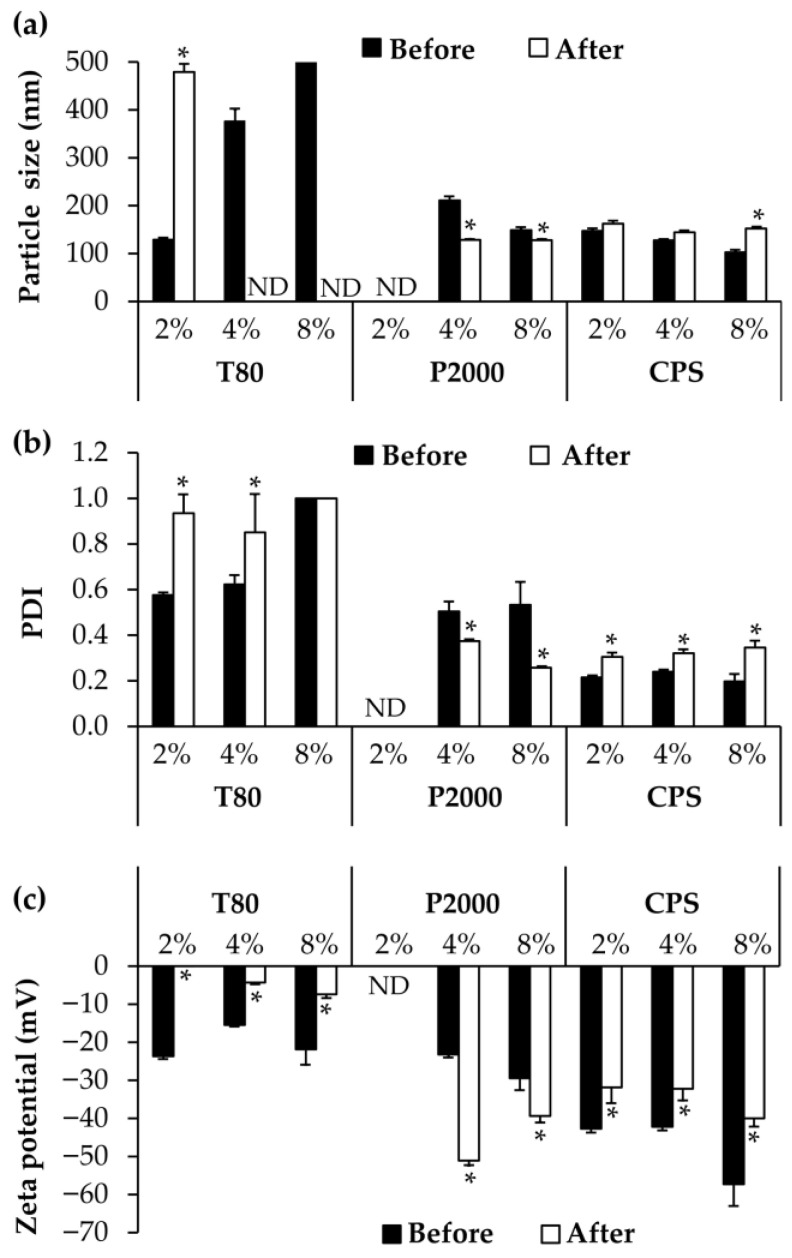
Particle size (**a**), polydispersity index (PDI) (**b**), and zeta potential (**c**) of NLCs developed using different types of surfactants, including Tween^®^ 80 (T80), Plantacare^®^ 2000 (P2000), and Coco Pea.Soft^®^ (CPS), at the various concentration of 2, 4 and 8% *w*/*w*. An asterisk (*) indicates significant differences in the particle size, PDI, and zeta potential of NLCs before and after the stability test, analyzed using a paired sample *t*-test (*p* < 0.05). ND = not determined.

The stability outcomes of the NLCs, as shown in Figure 2, backed up the prediction that the systems of Tween^®^ 80 were likely to be unstable because of their board PDI and weak apparent zeta potential values. The results demonstrated that Tween^®^ 80 yielded an unstable NLC with the enlarged particle exceeding the detection limit of Zetasizer Nano ZS, while the PDI increased to the maximum value of 1.0, which represented a highly polydisperse sample with multiple particle size populations [32]. Furthermore, the zeta potential values were significantly reduced to less than −10 mV. In contrast to Tween^®^ 80, the stable NLCs were developed using Plantacare^®^ 2000 and Coco Pea.Soft^®^. Despite the fact that the PDI and zeta potential values of those NLCs changed significantly after the heating and cooling cycles, both PDI and zeta potential values remained within or nearly within the acceptable ranges [32]. The PDI values ranged from 0.257 ± 0.1 to 0.374 ± 0.04, whereas the zeta potential values were more pronounced than −30 mV. 

Regarding these results, 2% *w*/*w* Coco Pea.Soft^®^ was suggested for the development of NLCs since it could produce NLC systems with a small particle size of 147.1 ± 2.8 nm, a narrow PDI of 0.215 ± 0.006, a pronounced zeta potential value of −42.7 ± 1.0 mV and be stable after the stability test even though the surfactant concentration was the lowest. Aside from the nanoscale of NLC particles, the distribution of particle sizes, essentially depicted by PDI, is an indication of their quality [32]. Different values were set for the acceptance criteria for the PDI; for example, PDI values below 0.2 are typically regarded as acceptable in practice for polymer-based nanoparticle, whereas PDI values below 0.3 are considered acceptable and signify a homogeneous phospholipid vesicle [33,34,35]. On the other hand, a zeta potential of at least −20 mV was suggested for sterically stabilized systems and −30 mV for electrostatic systems [36]. As a result, the NLC with 2% *w*/*w* Coco Pea.Soft^®^ was both acceptable in terms of particle size uniformity and achieved physical stability due to a strongly pronounced zeta potential value.

**Figure 3:**
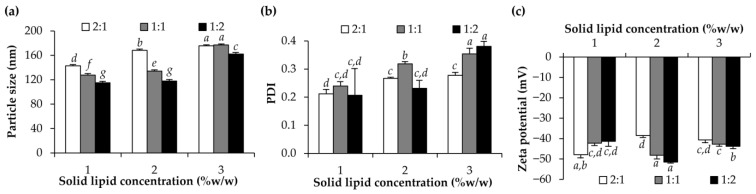
Particle size (**a**), polydispersity index (PDI) (**b**), and zeta potential (**c**) of NLCs developed using amount of lipid solid at the various concentration of 1, 2 and 3% *w*/*w* and ratio of solid lipid and liquid lipid was 2:1, 1:1, and 1:2. Different letters, *a*, *b*, *c*, *d*, *e*, *f*, and *g*, denote significant differences in the particle size, PDI, and zeta potential of NLCs analyzed using one-way ANOVA with post hoc Tukey test (*p* < 0.05).

Apart from the effects of surfactant types and amounts on the NLCs, the amount of solid lipid and liquid lipid were also investigated, and the results are shown in Figure 3. It was obviously shown that higher concentrations of the lipid phase resulted in larger particle sizes and lower PDI, which was straightforward. In addition, a higher proportion of liquid lipid in the lipid phase tended to reduce the particle size of the NLCs. The results were in line with those of Gokce et al. (2012) reported that NLCs with the same total lipid concentrations were smaller than solid lipid nanoparticles (SLN) under identical conditions [37]. Therefore, the presence of liquid lipid is one of the factors responsible for the smaller particle size of lipid nanoparticles. However, there was no correlation between the zeta potential of each formulation and the amount of lipid present. Although there were variances in zeta potential amongst the samples, they were all large in magnitude, which was in the range of −38.5 ± 1.2 to −51.5 ± 1.0 mV. The zeta potential is a fundamental parameter for electrokinetic potential for the thermodynamic stability of the colloidal systems and a high magnitude of the zeta potential from zero represents the ability of the particles to maintain a colloidal dispersion without coalescence, aggregation, or sedimentation [38].

**Figure 4:**
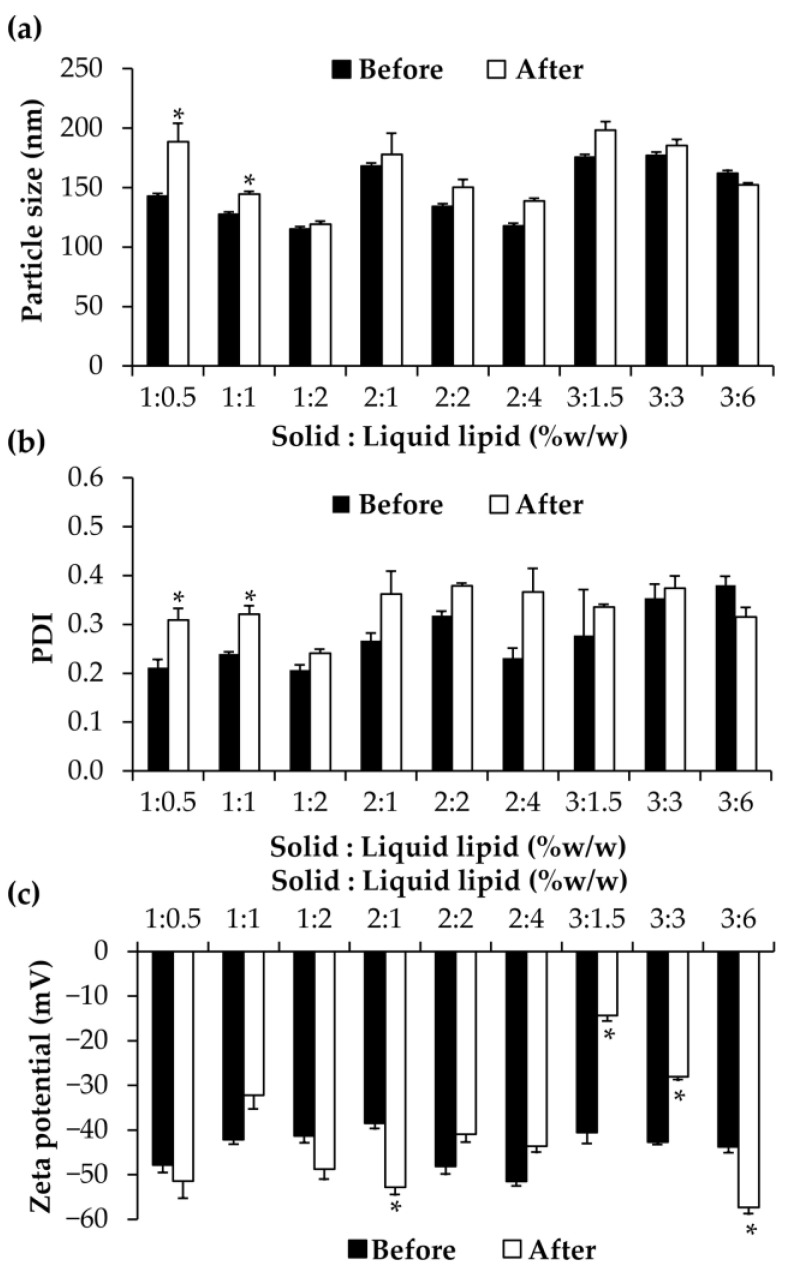
Particle size (**a**), polydispersity index (PDI) (**b**), and zeta potential (**c**) of NLCs developed using various amounts of solid lipid of 1, 2, and 3% *w*/*w*, and the ratio of solid lipid and liquid lipid was 2:1, 1:1, and 1:2. An asterisk (*) indicates significant differences in the particle size, PDI, and zeta potential of NLCs before and after the stability test, analyzed using a paired sample *t*-test (*p* < 0.05).

After the stability test in heating and cooling conditions, all NLCs had the same external appearance. However, the particle size, PDI, and zeta potential changed somewhat in some formulations, as shown in Figure 4. Nevertheless, the particle size of all NLCs was not greater than 200 nm. The NLC with solid lipid and liquid lipid ratios of 1:2 was found to be the most appropriate formulation since it had the smallest particle size both before and after the stability test. Furthermore, its PDI remained the narrowest and did not change after being stored in the heating and cooling conditions. Its zeta potential was also pronounced. Therefore, it was selected for further incorporation of CE0.5, the glutinous rice husk extract with anti-aging properties. Furthermore, the NLCs with solid lipid and liquid lipid ratios of 1:1 and 2:4 were also selected for further study due to their small particle size.

### 3.2. Glutinous Rice-Husk-Extract-Loaded NLCs

**Figure 5:**
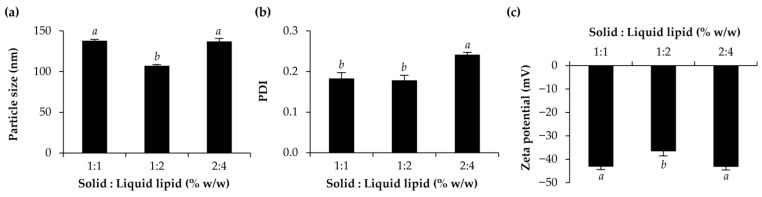
Particle size (**a**), polydispersity index (PDI) (**b**), and zeta potential (**c**) of glutinous rice-husk-extract-loaded NLCs using various amounts of solid lipid and liquid lipid. Different letters, *a*, *b* denote significant differences in the particle size, PDI, and zeta potential of NLCs analyzed using one-way ANOVA with post hoc Tukey test (*p* < 0.05), respectively.

The CE0.5 was incorporated into the selected NLCs with the solid lipid and liquid lipid ratios of 1:1 (NLC1), 1:2 (NLC2), and 2:4 (NLC3). Their particle size, PDI, and zeta potential are shown in Figure 5. The results were in line with the NLCs formulation without the extract, which showed that the formulation with solid lipid and liquid lipid ratios of 1:2 had the significantly smallest particle size (107.3 ± 1.3 nm) and the narrowest PDI (0.178 ± 0.012). When comparing NLCs with the same solid lipid concentration (1% *w*/*w*) but varied liquid lipid quantities (1 and 2% *w*/*w*), it was shown that the particle size and PDI of the NLCs tended to decrease with increasing liquid lipid proportion in the lipid phase. On the other hand, when comparing NLCs with the same solid-to-liquid lipid ratio (1:2) but varying lipid phase amounts, the higher concentration of the lipid phase resulted in a significantly larger particle size and border PDI value. However, a prominent zeta potential value in the range of −36.6 ± 0.2 to −43.3 ± 1.3 mV was detected in all glutinous rice-husk-extract-loaded NLCs. It could be predicted that all NLCs with CE0.5 were stable since a zeta potential greater than 30 mV typically maintained a stable system [38].

### 3.3. Entrapment Efficiency of Glutinous Rice-Husk-Extract-Loaded NLCs

**Figure 6:**
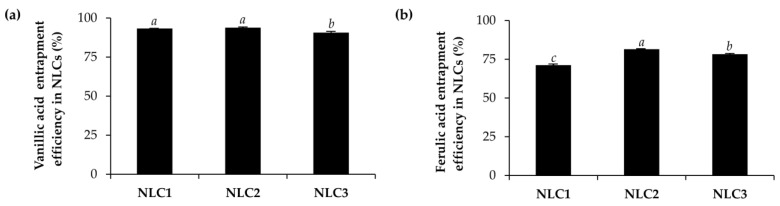
The entrapment efficacy of vanillic acid (**a**) and ferulic acid (**b**) from CE0.5-loaded NLCs (NLC1, NLC2, and NLC3). Different letters, *a*, *b* and *c*, denote significant differences in the entrapment efficacy of CE0.5-loaded NLCs analyzed using one-way ANOVA with post hoc Tukey test (*p* < 0.05).

The entrapment efficiency of bioactive compounds within NLCs is a crucial parameter that determines the effectiveness of the carrier system in delivering the compounds to the target site. In this study, the entrapment efficiency of two specific compounds, vanillic acid and ferulic acid, within NLCs was investigated and the results are shown in Figure 6. The findings clearly demonstrated that both vanillic acid and ferulic acid can be successfully entrapped within the NLCs. The entrapment efficiencies for vanillic acid ranged from 90.6 ± 0.6% to 93.8 ± 0.1%, indicating a high degree of encapsulation. On the other hand, the entrapment efficiencies for ferulic acid were slightly lower, ranging from 71.2 ± 0.1% to 81.5 ± 0.1%. The observed variations in entrapment efficiencies can be attributed to several factors, including the physicochemical properties of the compounds and the composition of the NLCs. Vanillic acid and ferulic acid possessed distinct molecular structures and physicochemical characteristics, which could influence their interactions with the lipid matrix of the NLCs. It is possible that vanillic acid exhibits a higher affinity for the lipids used in the NLC formulation, leading to its higher entrapment efficiency compared to ferulic acid. Furthermore, potential oxidation of ferulic acid to vanillic acid is indeed another factor that can contribute to the observed variations in entrapment efficiencies between the two compounds [39]. Furthermore, optimizing the compositions of NLCs can aid in designing effective NLC systems for the encapsulation and delivery of bioactive compounds [40]. Among all NLCs investigated, NLC2 showed the highest entrapment efficiency for the bioactive components of the glutinous rice husk extract, making it a promising candidate for further studies and applications.

### 3.4. Stability of Glutinous Rice-Husk-Extract-Loaded NLCs

**Figure 7:**
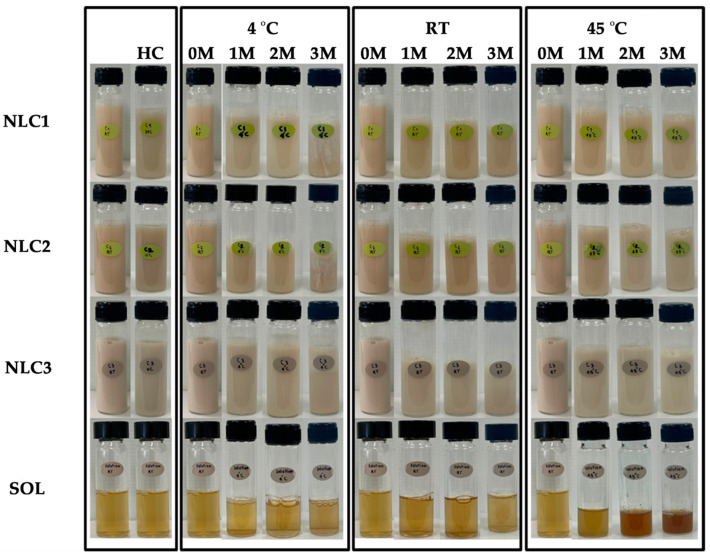
Physical appearance of glutinous rice husk extract (CE0.5)-loaded NLCs (NLC1, NLC2, and NLC3) and aqueous solution (SOL) before, after 8 heating–cooling cycles (HC), and after long-term storage in 4 °C, room temperature (RT), and 45 °C for 1, 2, and 3 months.

**Figure 8:**
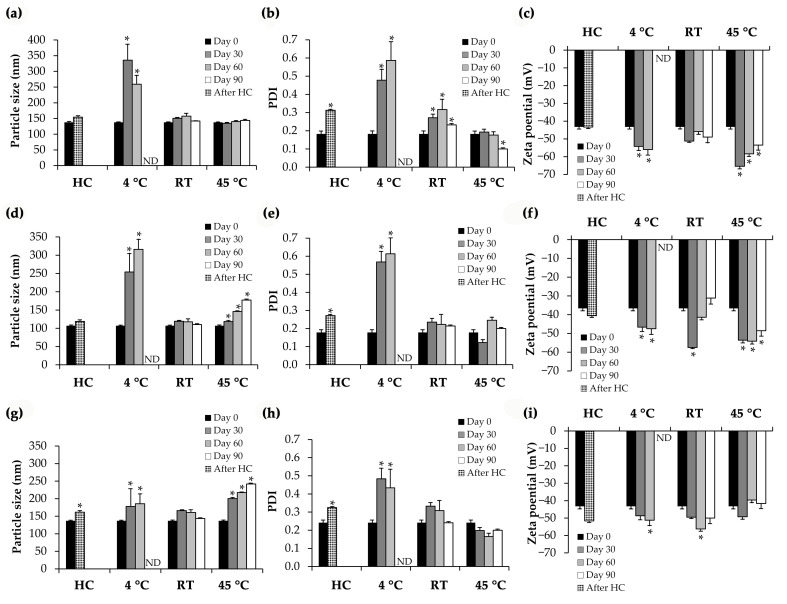
Particle size (**a**), polydispersity index (PDI) (**b**), and zeta potential (**c**) of glutinous rice husk extract (CE0.5)-loaded NLC1, particle size (**d**), PDI (**e**), and zeta potential (**f**) of CE0.5-loaded NLC2, and particle size (**g**), PDI (**h**), and zeta potential (**i**) of CE0.5-loaded NLC3 before (Day 0), after 8 heating–cooling cycles (HC), and after long-term storage in 4 °C, room temperature (RT), and 45 °C for 1, 2, and 3 months. Asterisk (*) denotes significant differences in the particle size, PDI, and zeta potential of NLCs analyzed using pair-sample *t*-test with Day 0 (*p* < 0.05). ND = not determined.

The external appearance of an aqueous solution and NLCs containing CE0.5 demonstrated that all NLCs were opaque liquids with a pale yellow color, which resembled the color of the CE0.5 aqueous solution as shown in Figure 7. However, NLC3, which contained the highest concentration of lipid phase, had the palest color. All NLCs were physically stable after 8 heating–cooling cycles and 3 months of long-term storage at various temperatures, except for the low temperature (4 °C). The particle size and PDI (Figure 8) strongly supported the instability at low temperatures because both the particle size and PDI of all NLCs stored at 4 °C have dramatically increased since the first month and have continuously increased, resulting in phase instability. The results were in line with the previous study, which stated that aggregation of the nanoparticles, which results in the production of larger particles, is an indication of instability [41].

In contrast, an aqueous solution of CE0.5 was found to have sedimented since the first month, and the color of the solution turned darker after being kept at a high temperature (45 °C) for 1 month, and continuously darker after the longer storage time. However, incorporation the CE0.5 in NLCs resulted in physical stability to the high temperature. However, incorporating CE0.5 into NLCs made the extract stable at high temperatures without sedimentation or color change. It is therefore possible to draw the conclusion that NLCs can improve the physical stability of CE0.5, particularly when exposed to high temperatures. However, low temperatures were not recommended for the storage of NLCs due to the phase separation occurring after 3 months of storage.

**Figure 9:**
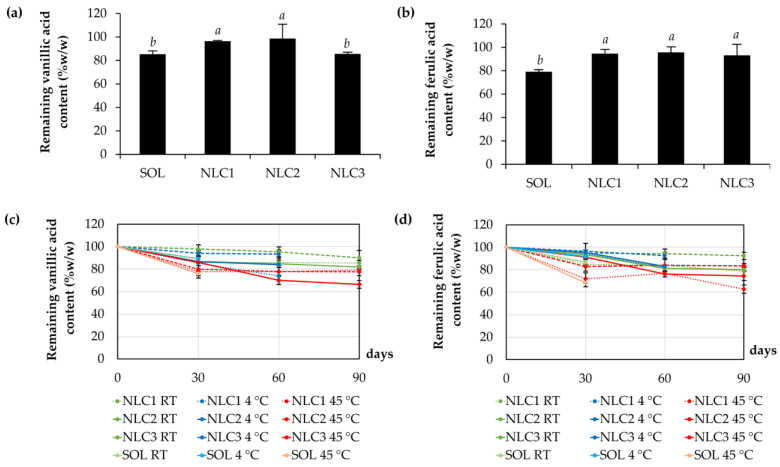
The remaining content of vanillic acid (**a**) and ferulic acid (**b**) after 8 cycles of heating–cooling conditions and the remaining content of vanillic acid (**c**) and ferulic acid (**d**) after long-term storage for 3 months (90 days) in room temperature (green), 4 °C (blue), and 45 °C (red) of NLC1, NLC2, NLC3, and aqueous solution (SOL). Different letters, *a* and *b*, denote significant differences in the content of vanillic and ferulic acid analyzed using one-way ANOVA with post hoc Tukey test (*p* < 0.05).

Aside from the enhancement of the physical stability of CE0.5, NLCs also prevent the degradation of the major bioactive compounds in CE0.5. The present study revealed that both vanillic and ferulic acid in CE0.5 aqueous solution deteriorated to 85.3 ± 2.8 and 79.2 ± 1.8% from the starting points, respectively, following eight cycles of heating–cooling conditions as shown in Figure 9. However, NLCs can diminish the degradation of both compounds. The NLCs significantly enhanced the vanillic and ferulic acid content to more than 95% after the accelerated stability test, except for NLC3, which could protect only ferulic acid from the degradation. This finding highlights the importance of formulation optimization and the need to consider the compatibility between the bioactive compounds and the NLC components to achieve optimal stability. By selecting suitable components and adjusting their ratios, it is possible to enhance stability, protect the bioactive compounds from degradation, and maximize the potential benefits of NLCs in various applications.

On the other hand, NLCs also enhance the stability of CE0.5 in a long-term study. Since the CE0.5 aqueous solution sedimented after one month at all temperatures, including 4 °C, room temperature (30 °C), and 45 °C, it was excluded from further investigation. However, the results noted that both vanillic and ferulic acids degraded after one month of storage, especially at high temperatures. Vanillic acid was found to be reduced to less than 80% (~20% degradation), whereas ferulic acid was found to be reduced to less than 70% (~30% degradation) after being kept at 45 °C for 1 month. The findings were consistent with the previous study, which showed that vanillic acid decomposed at high temperatures, decaying 15% at 60 °C, 25% at 80 °C, and 37% at 100 °C [42]. The protective effect of NLCs is primarily attributed to their ability to act as a physical barrier [43]. The lipid matrix of NLCs serves as a protective shell, shielding the bioactive compounds from external factors such as heat and other degrading agents. This physical barrier prevents direct contact and interaction between the compounds and the degrading factors, thereby preserving their stability.

### 3.5. Release of Glutinous Rice-Husk-Extract-Loaded NLCs

**Figure 10:**
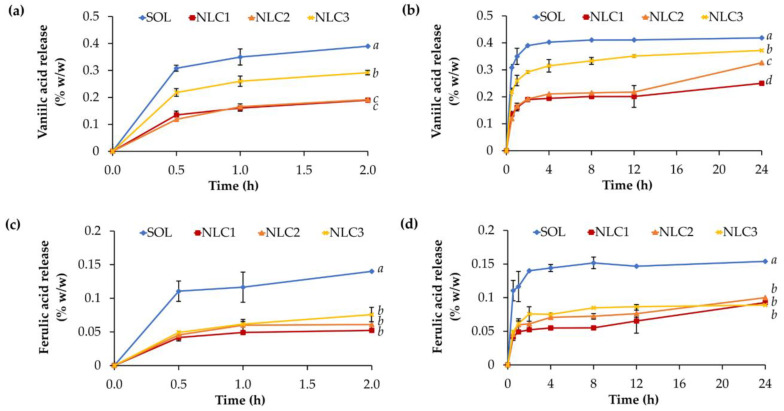
Release profiles of vanillic acid magnified between 0 and 2 h (**a**) and throughout 24 h (**b**) as well as ferulic acid magnified between 0 and 2 h (**c**) and throughout 24 h (**d**) from CE0.5-loaded NLCs (NLC1, NLC2, and NLC3) and aqueous solution (SOL). Different letters, *a*, *b*, *c*, and *d*, denote significant differences in the release amount of vanillic and ferulic acid analyzed using one-way ANOVA with post hoc Tukey test (*p* < 0.05).

The release pattern of vanillic and ferulic acid from NLCs, as shown in Figure 10, highlights that their release follows a controlled and sustained pattern with a lower release amount compared to the solution. The likely explanation was due to the lipid matrix of NLCs, which provides a barrier to slow down the diffusion of the encapsulated compounds, resulting in a gradual release over time [44]. The assumption that both vanillic acid and ferulic acid were entrapped within the NLCs rather than being deposited on the surface was based on the results of the stability test, which showed a decrease in both compounds over time when they were not entrapped in the NLCs (Figure 9). This controlled release profile is beneficial for maintaining a prolonged therapeutic effect and minimizing potential side effects [45]. However, it is possible that some vanillic acid and ferulic acid were deposited on the surface of the NLCs, which resulted in a burst release within the first 30 min, as shown in Figure 10. Nevertheless, for a more comprehensive understanding of the NLC system, it has been suggested that an analysis of the internal structure (e.g., small-angle X-ray scattering (SAXS) profile) and morphology (e.g., transmission electron microscopy (TEM), cryo-TEM, etc.) be conducted in future studies.

The present study noted that the release amount of vanillic acid from NLCs was higher compared to ferulic acid. This observation can be attributed to the higher entrapment efficiency of vanillic acid in the NLCs. The entrapment efficiency refers to the percentage of the compound successfully encapsulated within the NLCs. A higher entrapment efficiency indicates a larger proportion of the compound retained within the lipid matrix of the NLCs. Consequently, during the release process, a larger amount of vanillic acid is gradually released from the NLCs due to its higher initial entrapment. On the other hand, other factors such as the solubility, molecular weight, and interactions of the compounds with the lipid matrix can also influence the release rate [46].

### 3.6. Skin Permeation and Skin Retention of Glutinous Rice-Husk-Extract-Loaded NLCs

**Figure 11:**
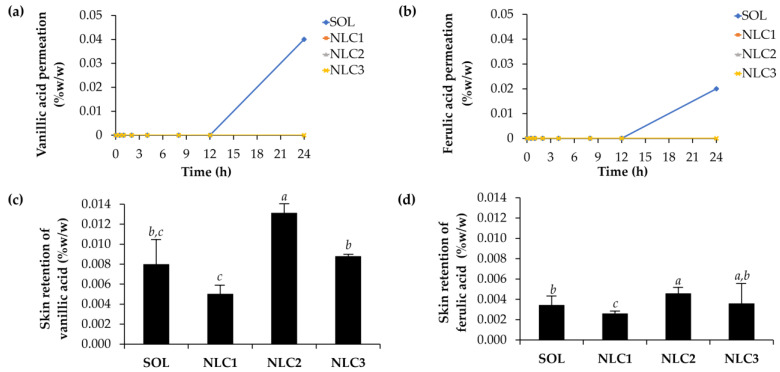
Skin permeation of vanillic acid (**a**) and ferulic acid (**b**), as well as skin retention of vanillic acid (**c**) and ferulic acid (**d**) from CE0.5 loaded NLCs (NLC1, NLC2, and NLC3) and aqueous solution (SOL) after 24 h of application. Different letters, *a*, *b*, and *c*, denote significant differences in the skin retention of vanillic and ferulic acid analyzed using one-way ANOVA with post hoc Tukey test (*p* < 0.05).

The skin permeation and skin retention profiles of vanillic and ferulic acid from CE0.5-loaded NLCs are shown in Figure 11, emphasizing a comparison with the solution. It was obvious that both vanillic and ferulic acid from the solution were able to permeate through the skin and reach the receptor chamber of the Franz diffusion cell. In contrast, no skin permeation was observed for the NLC formulations. The results were in line with the release profiles of vanillic and ferulic acid. As both acids were released from the solution at higher rates than NLCs (Figure 10), they have the ability to penetrate the epidermal layer. In contrast, NLCs gradually release a modest amount of vanillic acid and ferulic acid. Furthermore, NLCs with higher lipophilicity were able to remain in the skin layer, whereas aqueous solutions were compatible with viable epidermis and dermis cells, resulting in enhanced acid penetration, especially after long durations, e.g., 24 h. Permeation through the skin can result in the systemic absorption of bioactive compounds, which may not be desirable for cosmetic products intended for topical use [47]. This can increase the risk of systemic side effects or interactions with other medications. To address these concerns, cosmetic formulations often employ strategies to enhance localized effects and minimize systemic absorption. Controlled-release systems, such as NLCs, are specifically designed to prolong the residence time of bioactive compounds on the skin, allowing for gradual release and reducing systemic absorption [48]. This approach helps mitigate the disadvantages associated with direct permeation through the skin and enhances the targeted localized effects of cosmetic products.

In contrast to skin permeation, NLCs exhibited higher skin retention compared to the solution formulation. This can be attributed to several factors associated with NLCs, including their ability to enhance adhesion, control release of bioactive compounds, improve penetration into the skin, and provide protection from external factors [49,50]. These combined mechanisms contribute to the superior skin retention observed with NLCs. Among all NLC formulations, NLC2 demonstrated the highest delivery of both vanillic and ferulic acid into the skin layer. The likely explanation was due to its smallest particle size. As NLC2 had the smallest particle size of 107.3 ± 1.3 nm it could easily penetrate the skin barrier and reach the deeper layers. The small particle size of NLC2 increases the surface area available for interaction with the skin, facilitating the efficient delivery of vanillic and ferulic acid [51]. Additionally, the small particle size may contribute to a higher degree of skin adhesion, ensuring prolonged contact and increased absorption of the bioactive compounds [49]. Thus, the smaller particle size of NLC2 likely played a crucial role in its superior delivery of vanillic and ferulic acid into the skin layer compared to other NLC formulations.

## 4. Conclusions

The present study provides valuable insights into the use of NLCs as a promising approach to enhance the skin delivery and stability of vanillic acid and ferulic acid derived from glutinous rice husk extract. It was remarkable that NLCs effectively encapsulate and protect the bioactive compounds, leading to higher entrapment efficiencies compared to a solution. The controlled and sustained release pattern of vanillic acid and ferulic acid from NLCs suggests their potential for prolonged and targeted delivery. The findings also highlight the superior skin retention capabilities without the permeation of NLCs compared to the solution. Notably, NLC2 demonstrated the highest delivery into the skin layer, which can be attributed to its smaller particle size, facilitating enhanced skin delivery. This research emphasizes the promising application of NLCs in enhancing the delivery and stability of bioactive compounds in cosmetic formulations and related fields. Further investigation of in vitro toxicity and clinical evaluation were suggested for further investigation to ensure the safety, performance, and efficacy of the NLCs.

## Data Availability

Data available on request.
